# Optimum programmed intermittent epidural bolus interval time of ropivacaine 0.0625% with dexmedetomidine 0.4 μg/ml at a fixed volume of 10 mL: a randomized controlled trial

**DOI:** 10.3389/fphar.2024.1368222

**Published:** 2024-03-26

**Authors:** Zhong Mei, Qingtao Wang, Shaobo Song, Wenying Lu, Jing Yu

**Affiliations:** Department of Anesthesiology, Affiliated Xiaoshan Hospital, Hangzhou Normal University, Hangzhou, China

**Keywords:** EI90, PIEB, dexmedetomidine, ropivacaine, epidural labor analgesia

## Abstract

**Background:**

The aim of our study was to administer adequate local anesthetic in programmed intermittent epidural bolus (PIEB) to avoid breakthrough pain and decrease the use of manual and PCEA boluses. We, therefore, conducted this study to determine the effective PIEB interval time between boluses of ropivacaine 0.0625% with dexmedetomidine 0.4 μg/ml at a fixed volume of 10 mL in 90% of subjects (EI_90_), without the use of patient-controlled epidural analgesia (PCEA).

**Methods:**

A total of 80 subjects were included in the final statistical analysis from 23 August 2022 to 22 November 2022. The subjects were randomly assigned to one of four different PIEB time intervals: 40, 50, 60, and 70 min (groups 40, 50, 60, and 70), respectively. The primary outcome was the effective epidural labor analgesia, defined as no use of PCEA bolus or a manual bolus until the end of the first stage of labor or within 6 hours after loading dose administration. The PIEB EI_90_ (95% CI) between boluses of ropivacaine 0.0625% with dexmedetomidine 0.4 μg/ml at a fixed volume of 10 mL was estimated using probit regression.

**Results:**

The effective PIEB interval time between boluses of ropivacaine 0.0625% with dexmedetomidine 0.4 μg/ml at a fixed volume of 10 mL in 90% of subjects without the use of PCEA was 45.4 (35.5–50.5) minutes using probit regression. No statistical differences were found in the proportion of subjects with Bromage score > 0, hypotension, pruritus, nausea, and vomiting between groups. However, the highest sensory block (pinprick) in the 40-min group was significantly higher than that in the other groups.

**Conclusion:**

The estimated value for EI_90_ for PIEB between boluses of ropivacaine 0.0625% with dexmedetomidine 0.4 μg/ml at a fixed volume of 10 mL using probit regression was 45.4 (35.5–50.5) minutes. Furthermore, future studies are warranted to be established to determine the optimal parameters for different regimens in clinical practice.

## 1 Introduction

Programmed intermittent epidural bolus (PIEB) is an advanced labor analgesia technique that provides automated epidural administration at a fixed local anesthetic volume and a set interval. Compared with the continuous epidural infusion technique (CEI), PIEB can improve maternal satisfaction, reduce the incidence of breakthrough pain, and produce a more uniform spread of the epidural local anesthetic (McKenzie CP et al., 2016; Roofthooft E et al., 2020). The patient-controlled epidural analgesia (PCEA) technique allows subjects to autonomously manage different stages of breakthrough pain. Therefore, PIEB combined with PCEA is one of the optimum methods to maintain epidural labor analgesia to date.

Recently, dexmedetomidine has been advocated as a potentially ideal adjuvant for epidural labor analgesia instead of opioids, with the advantage of a lower incidence of pruritus without influencing maternal satisfaction and the quality of labor analgesia (Zhou H et al., 2022; Lao C et al., 2023). Furthermore, our subsequent unpublished study (ChiCTR2200062309; https://www.chictr.org.cn/bin/project/edit?pid=159765) and one other study demonstrated that epidural dexmedetomidine was non-inferior to fentanyl in terms of hourly ropivacaine consumption for labor analgesia (Pang RY et al., 2022). So far, the optimum PIEB interval time when dexmedetomidine is added to local anesthetic remains unknown.

We, therefore, conducted this study to determine the effective PIEB interval time between boluses of ropivacaine 0.0625% with dexmedetomidine 0.4 μg/ml at a fixed volume of 10 mL in 90% of subjects (EI_90_) without the use of PCEA. We hypothesized that the effective PIEB interval time between boluses of 10 ml ropivacaine 0.0625% with dexmedetomidine 0.4 μg/ml would be between 40 and 70 min.

## 2 Materials and methods

### 2.1 Design and study subjects

The randomized, interval time-finding study was approved by the Research Ethics Board on 22 August 2022 (approval number: K2022040) and registered in the Chinese Clinical Trial Registry on 23 August 2022 (ChiCTR2200062918; https://www.chictr.org.cn/bin/project/edit?pid=177423). All subjects were informed that they were receiving an off-label use of dexmedetomidine in the neuraxial administration and provided written informed consent. The study was conducted from 23 August 2022 to 22 November 2022. We confirm that our study followed the Declaration of Helsinki and the Consolidated Standards of Reporting Trials (CONSORT) statement.

We enrolled term nulliparous subjects of the American Society of Anesthesiologists physical status (ASA) II, with singleton fetuses, regular uterine contractions of 3–5 min, and numerical rating scale (NRS) pain scores >3 at the time of labor analgesia request (scale: 0 = no pain, 10 = the worst pain). Exclusion criteria include contraindication to neuraxial analgesia, refusal to participate in the study, and allergy to ropivacaine or dexmedetomidine.

### 2.2 Study protocol

The epidural was carried out by an attending anesthesiologist in the left lateral position at the estimated L2–3 or L3–4 interspace using a 17G Tuohy needle (Zhejiang Fert Medical Device Co., Ltd.; China) with the loss-of-resistance-to-air technique. A wire-reinforced epidural catheter was advanced into epidural space 3–5 cm and then secured. A test dose of 3 ml of 1% lidocaine was administered. After the safety assessment of the test dose at 5 min, a loading dose of 15 ml of ropivacaine 0.0625% with dexmedetomidine 0.4 μg/ml was administered. Then, the PIEB infusion pump (REHN11; Jiangsu Renxian Medical Technology Co., Ltd.) was connected to the epidural catheter and programmed to administer the first bolus of 10 ml 1 h after the loading dose. Subsequent boluses were administered at a PIEB time interval.

Subjects with a NRS pain score ≤3 at 30 min after the end of the loading dose infusion were entitled to continue in the study. If the NRS pain score was still >3 at 30 min, the loading dose was declared a failure for research purposes, and the subject was withdrawn from the study. Randomization was conducted by an assistant who was not further involved in the study. A randomization code sequence was generated by MedCalc 18.2.1 (MedCalc Software BV, Ostend, Belgium). Based on the coding sequence in the successive envelopes, the subjects were randomly assigned to one of four different PIEB time intervals: 40, 50, 60, and 70 min (groups 40, 50, 60, and 70), respectively. The PIEB infusion pump parameters were set by a certified registered nurse anesthetist (CRNA) who was not involved in subsequent experimental steps. The attending anesthesiologists, other investigators, and subjects participating in the study were blinded to the parameters of the pump. The other parameters of the PIEB pump were as follows: the PIEB infusion pump initiated and delivered boluses of 10 mL of 0.0625% ropivacaine combined with 0.4 μg/ml dexmedetomidine at one of four different PIEB time intervals post-loading dose and was programmed to deliver boluses at 300 mL/h. Subjects could add 10 mL PCEA boluses by themselves with a lockout time of 15 min and a maximum dose of 35 mL/h if they felt uncomfortable. If the subject pushed the PCEA button or requested a manual bolus, the case was considered a failure of PIEB interval time.

The primary outcome was the effective epidural labor analgesia, defined as no use of PCEA bolus or a manual bolus until the end of the first stage of labor or within 6 h after loading dose administration.

The fetal heart rate was continuously monitored during labor analgesia. The baseline systolic blood pressure was recorded as the mean of three consecutive systolic blood pressure measurements before labor analgesia. All assessments, including pain scores, sensory block to pinprick, motor block according to the modified Bromage score (3 = inability to flex ankles; 2 = inability to flex knees but able to flex ankles; 1 = inability to raise extended legs but able to flex knees; 0 = able to raise the extended leg) (Gabriel L et al., 2019), noninvasive blood pressure, pulse oximetry, and maternal heart rate, were performed by a blinded assistant every 10 min until 30 min after the loading dose and then every hour until the end of the study. The secondary outcomes were duration of the stage of labor, Apgar scores, hypotension (defined as a 20% drop or more from the baseline systolic blood pressure), bradycardia (defined as heart rate <60 bpm), pruritus, nausea, and vomiting. Additional data included age, height, weight, gestational age, nulliparous, spontaneous or induced labor, oxytocin administration, cervical dilation, and NRS pain scores.

### 2.3 Statistical analysis

Based on our pre-study in which the proportions of subjects with effective epidural labor analgesia were 1.0, 0.8, 0.5, and 0.4 in subjects who were randomly assigned to 40, 50, 60, and 70 min intervals, respectively. Ten subjects per subgroup were required at a power of 0.90 and a significance level of 0.05 (the Cochran–Armitage test for trend in proportions, PASS 11). We increased the sample size to 20 per subgroup to account for dropouts.

Epidural labor analgesia was effective if no PCEA or manual bolus was used until the end of the first stage of labor or within 6 h after the loading dose. The PIEB EI_90_ value (95% CI) between boluses of ropivacaine 0.0625% with dexmedetomidine 0.4 μg/ml at a fixed volume of 10 mL was estimated using probit regression.

Statistical analyses were performed using SPSS 25.0 (IBM Corp, Armonk, NY). The Shapiro–Wilk test was used to assess the normal distribution of the data. Continuous variables with a normal distribution were shown as mean (±SD) and compared using one-way ANOVA. Continuous variables with non-normal distribution were shown as median (with quartiles) and compared using the Mann–Whitney U test, as appropriate. Categorical variables were shown as numbers (*n*, %) and compared using the chi-squared test or Fisher’s exact test. *p* < 0.05 was defined as statistically significant.

## 3 Results

**FIGURE 1:**
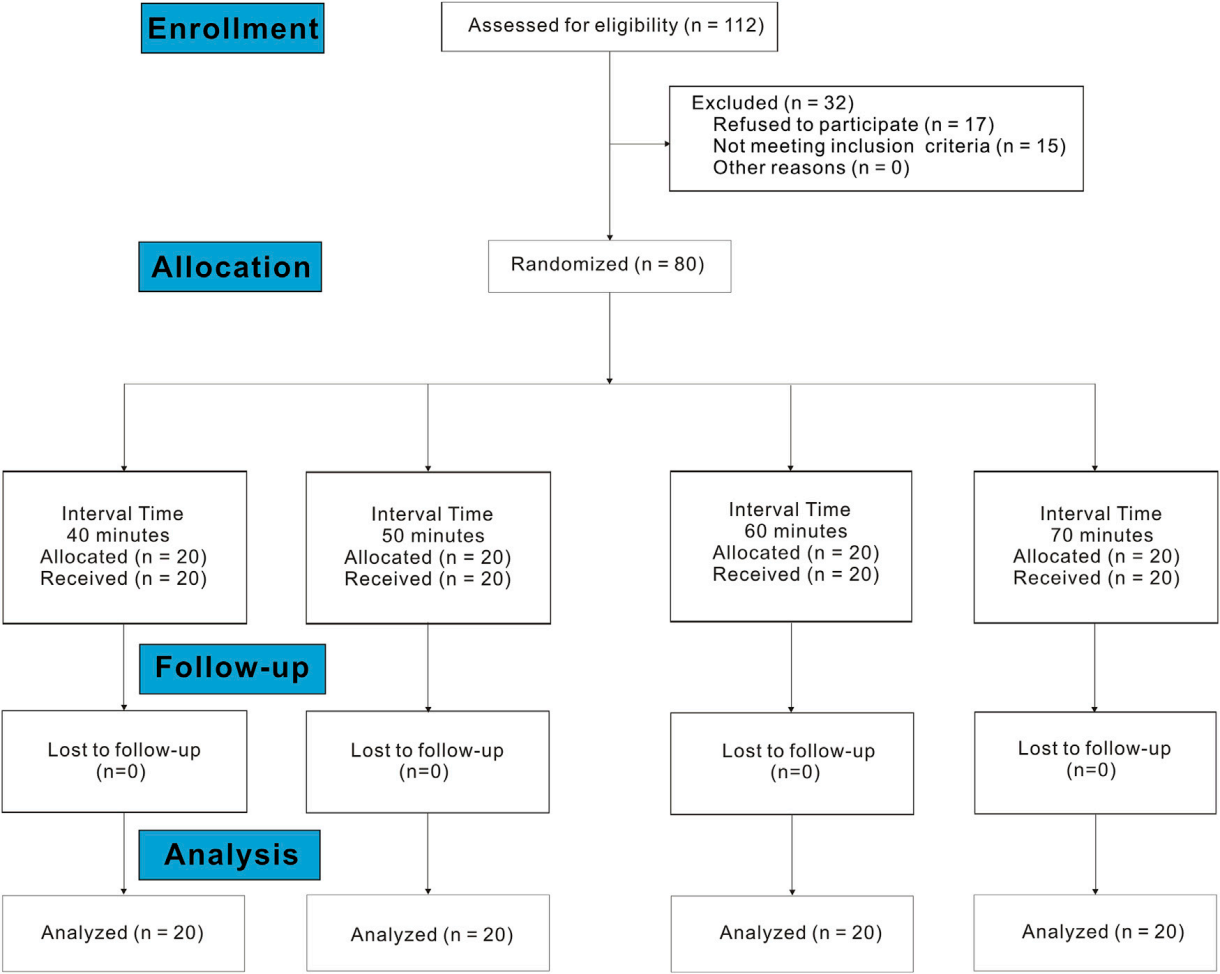
Consolidated Standards of Reporting Trials flow diagram.

A total of 112 subjects were assessed for eligibility in the study between 23 August 2022 and 22 November 2022. Of these, 17 refused to participate, and 15 did not meet the inclusion criteria. Finally, data from 80 subjects were included in the data analysis (Figure 1). The demographic data on subjects are shown in Table 1. There were no statistically significant differences between the groups in age, height, weight, gestational age, cervical dilation at request, cervical dilation at the end of the study, NRS before epidural analgesia, proportion of subjects using oxytocin, and proportion of subjects with spontaneous or induced labor.

**TABLE 1:** Demographic data.

	Interval time, 40-min (*n* = 20)	Interval time, 50-min (*n* = 20)	Interval time, 60-min (*n* = 20)	Interval time, 70-min (*n* = 20)	*p*-value
Age (years)	28.3 ± 3.2	28.8 ± 2.9	26.4 ± 3.8	28.7 ± 4.2	0.121
Height (cm)	160.0 ± 6.0	161.6 ± 4.7	161.8 ± 4.1	161.0 ± 3.6	0.200
Weight (kg)	67.6 ± 9.5	63.9 ± 5.9	72.3 ± 11.5	67.2 ± 9.9	0.108
Gestational age (weeks)	39.2 ± 1.1	38.2 ± 2.7	38.7 ± 1.0	39.2 ± 1.1	0.232
Nulliparous	12 (60%)	15 (75%)	13 (65%)	13 (65%)	0.916
Spontaneous labor	19 (95%)	17 (85%)	18 (90%)	18 (90%)	0.740
Induced labor	1 (5%)	3 (15%)	2 (10%)	2 (10%)	0.740
Oxytocin administration	4 (20%)	4 (20%)	7 (35%)	5 (25%)	0.491
Cervical dilation at request (cm)	3 (2–3)	3 (2–3)	2 (2–3)	2 (2–3)	0.719
Cervical dilation at the end of the study (cm)	10 (10–10)	10 (10–10)	10 (5–10)	10 (7–10)	0.331
NRS before epidural analgesia	5 (4–6)	6 (5–7)	5 (3–6)	5 (4–6)	0.069

Data are mean ± SD (standard deviation), or median (interquartile range) or number (%).

NRS pain scores, numerical rating scale pain scores.

**FIGURE 2:**
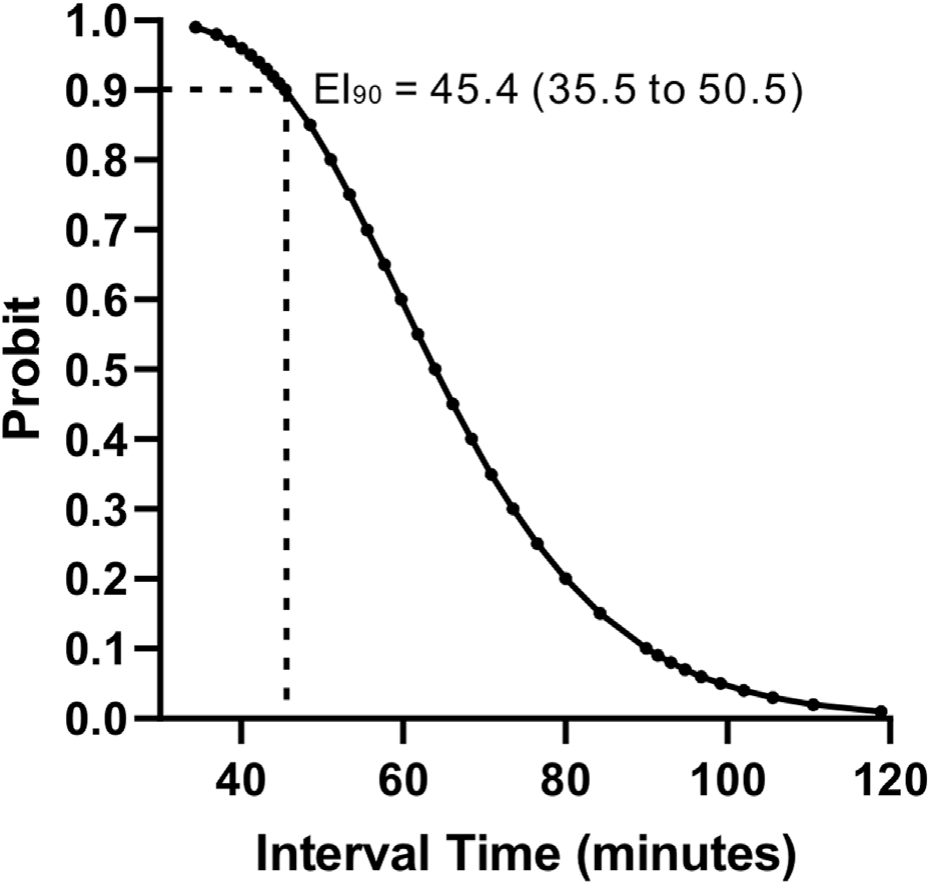
Interval–response curve for the effective PIEB interval time between boluses of ropivacaine 0.0625% with dexmedetomidine 0.4 μg/ml at a fixed volume of 10 mL without the use of PCEA derived from probit analysis. PIEB, programmed intermittent epidural bolus; PCEA, patient-controlled epidural analgesia.

The observed proportion of subjects with effective epidural labor analgesia at different PIEB interval times is shown in Table 2. Labor analgesia was effective in 95, 80, 70, and 30% of subjects of the 40-, 50-, 60-, and 70-min groups, respectively. The effective PIEB interval time between boluses of ropivacaine 0.0625% with dexmedetomidine 0.4 μg/ml at a fixed volume of 10 mL in 90% of subjects without the use of PCEA was 45.4 (35.5–50.5) minutes using probit regression (Figure 2).

**TABLE 2:** Observed proportion of subjects with effective epidural labor analgesia at different PIEB interval times.

Interval time (minutes)	Success	Number of patients	Effective analgesia rate (%)
40	19	20	95
50	16	20	80
60	14	20	70
70	6	20	30

Sensory block, motor block, and side effects for each PIEB interval time are presented in Table 3. No statistical differences were found in the proportion of subjects with Bromage score >0, hypotension, pruritus, nausea, and vomiting between the groups. However, the highest sensory block (pinprick) in the 40-min group was significantly higher than that in the other groups (*p* < 0.001).

**TABLE 3:** Sensory block, motor block, and side effects.

	Interval time, 40-min (*n* = 20)	Interval time, 50-min (*n* = 20)	Interval time, 60-min (*n* = 20)	Interval time, 70-min (*n* = 20)	*p*-value
Highest sensory block (pinprick)	T9 (8–10)	T10 (10–10)*	T10 (10–10)*	T10 (10–10)*	0.001*
Bromage score > 0	0 (0%)	0 (0%)	0 (0%)	0 (0%)	>0.999
Hypotension	0 (0%)	0 (0%)	0 (0%)	0 (0%)	>0.999
Nausea and vomiting	0 (0%)	0 (0%)	0 (0%)	0 (0%)	>0.999
Pruritus	0 (0%)	0 (0%)	0 (0%)	0 (0%)	>0.999
Bradycardia	0 (0%)	0 (0%)	0 (0%)	0 (0%)	>0.999

Data are median (interquartile range) or number (%).

*p < 0.05 for comparison with the interval time of 40-min value using one-way analysis of variance (ANOVA) and the least significant difference (LSD) *post hoc* test.

## 4 Discussion

Our study showed that the effective PIEB interval time between boluses of ropivacaine 0.0625% with dexmedetomidine 0.4 μg/ml at a fixed volume of 10 mL in 90% of subjects without the use of PCEA was 45.4 (35.5–50.5) minutes.

The optimum PIEB interval time when dexmedetomidine is added to the local anesthetic for epidural labor analgesia remains unknown in clinical practice. At present, the PIEB technique has been advocated as one of the common methods for labor analgesia in numerous obstetric anesthesia units (Roofthooft E et al., 2020; Song Y et al., 2021). Studies have shown that dexmedetomidine was non-inferior compared with opioid adjuvant in terms of hourly local anesthetic consumption when administered epidurally (Pang RY et al., 2022). Additionally, dexmedetomidine might be a potentially ideal adjuvant for epidural labor analgesia instead of opioids, with the advantage of a lower incidence of pruritus without influencing maternal satisfaction and the quality of labor analgesia (Zhou H et al., 2022; Lao C et al., 2023). Therefore, we conducted this study to determine the effective PIEB interval time between boluses of ropivacaine 0.0625% with dexmedetomidine 0.4 μg/ml at a fixed volume of 10 mL in 90% of subjects (EI_90_) without the use of PCEA.

Similar to previous studies, we defined effective epidural labor analgesia as no use of PCEA bolus or a manual bolus until the end of the first stage of labor or within 6 h after loading dose administration (Epsztein Kanczuk M et al., 2017; Zakus P et al., 2018; Shatalin D et al., 2021). Some may dispute the definition of effective epidural analgesia. In this study, we purposed to explore the relevant parameters of PIEB technology and exclude the confounding effect of PCEA, whereas frequent PCEA dosing procedures may also lead to an increased rate of maternal dissatisfaction. Therefore, the aim of our study was to administer the adequate local anesthetic in PIEB to avoid breakthrough pain and decrease the use of manual and PCEA boluses.

Our study suggested that the optimum PIEB interval time was 45 min, which was similar to the reported interval of 35–41 min (Epsztein Kanczuk M et al., Song Y et al., 2022; Bittencourt R et al., 2019). Although different concentrations and drugs, pain thresholds, and bolus delivery rates were used between studies, we propose that a fixed volume and dose of local anesthetic plus adjuvant (volume ≥5 mL, dose ≥6.25 mg) would produce a more uniform spread of the epidural local anesthetic and lead to similar analgesic durations (a 35–45 min intervals) (Epsztein Kanczuk M et al., Song Y et al., 2022; Bittencourt R et al., 2019). This may be generalized to the PIEB technique in general, which requires a large-sample multicenter study to determine.

No subjects have experienced motor block in our study because we adopted an anesthesia regimen with an ultra-low concentration and a high volume of local anesthetic, which has been recommended by substantial documented studies (Campbell DC et al., 2000; Owen MD et al., 2002; Bolukbasi D et al., 2005; Beilin Y et al., 2007; Ginosar Y et al., 2010; Zhao B et al., 2019). More dilute and larger volumes of solutions (e.g., 0.0625% ropivacaine) can provide more effective labor analgesia, with the advantage of maintaining the ability to urinate and ambulate, while accelerating cervical dilation, shortening the labor process, and reducing the use of oxytocin (Campbell DC et al., 2000; Ginosar Y et al., 2010; Zhao B et al., 2019). Studies have demonstrated that 0.0625% ropivacaine and bupivacaine were equivalent and effective for epidural labor analgesia (Owen MD et al., 2002; Bolukbasi D et al., 2005; Beilin Y et al., 2007).

Our study showed that the shorter PIEB interval of 40 min may produce higher sensory block to pinprick than the longer PIEB intervals of 50–70 min, which was similar to the previous studies using different regimens (Epsztein Kanczuk M et al., Song Y et al., 2022; Bittencourt R et al., 2019). The PIEB technique with optimum interval time can produce a more uniform spread of epidural local anesthetic and reduce breakthrough pain.

Our study also has several limitations. First, dexmedetomidine has not been approved for clinical use by the United States Food and Drug Administration. Second, our results may be valid for the solution of ropivacaine 0.0625% with dexmedetomidine 0.4 μg/ml. If the ropivacaine concentration is higher, the duration of analgesia is usually extended, and the results of the study would certainly have been different. Third, effective epidural labor analgesia was defined as no use of PCEA bolus or a manual bolus until the end of the first stage of labor or within 6 h after loading dose administration. Therefore, our findings may only be applicable to epidural labor analgesia within 6 h after the loading dose. Fourth, the pain threshold NRS score was required to be controlled at or below three, whereas some other studies set the NRS pain score less than or equal to one as the control target. Our data may only be appropriate for pain threshold targets less than or equal to three. Lastly, a low proportion of patients in this study received oxytocin (most in spontaneous labor), and the median cervical dilation at the initiation of epidural analgesia was low (the latent phase of labor). Both of these factors influence the amount of drug necessary to treat the pain of labor. Women in more active labor or receiving oxytocin will require higher doses (and, therefore, perhaps a shorter interval).

## 5 Conclusion

In conclusion, the estimated value for EI_90_ for PIEB between boluses of ropivacaine 0.0625% with dexmedetomidine 0.4 μg/ml at a fixed volume of 10 mL using probit regression was 45.4 (35.5–50.5) minutes. Furthermore, a large-sample multicenter study is warranted to be established to confirm that a fixed volume and dose of local anesthetic with adjuvant (volume ≥5 mL, dose ≥6.25 mg) requires an interval of 35–45 min (EI_90_) for PIEB despite using different concentrations and drugs.

## Data Availability

The raw data supporting the conclusion of this article will be made available by the authors, without undue reservation.

## Abbreviations

PIEB, programmed intermittent epidural bolus; CEI, continuous epidural infusion; PCEA, patient-controlled epidural analgesia; EI_90_, effective interval in 90% patients; CONSORT, Consolidated Standards of Reporting Trials; ASA, American Society of Anesthesiologists; NRS pain scores, numerical rating scale pain scores; CRNA, certified registered nurse anesthetist; CI, confidence interval; ANOVA, analysis of variance.
